# Roughness Estimation and Image Rendering for Glossy Object Surface

**DOI:** 10.3390/jimaging11090296

**Published:** 2025-08-28

**Authors:** Shoji Tominaga, Motonori Doi, Hideaki Sakai

**Affiliations:** 1 Department of Computer Science, Norwegian University of Science and Technology, 2815 Gjovik, Norway; 2 Department of Business and Informatics, Nagano University, Ueda 386-1298, Japan; 3 Department of Communication Engineering, Osaka Electro-Communication University, Neyagawa 572-8530, Japan; doi@osakac.ac.jp; 4 Professor Emeritus, Graduate School of Informatics, Kyoto University, Kyoto 606-8501, Japan; hsakai@i.kyoto-u.ac.jp

**Keywords:** roughness estimation, glossy surface, dielectric objects, handcrafted lacquerwares, plastics, laser scanning system, Beckmann function, HDR images, physical surface roughness, roughness parameter

## Abstract

We study the relationship between the physical surface roughness of the glossy surfaces of dielectric objects and the roughness parameter in image rendering. The former refers to a measure of the microscopic surface structure of a real object’s surface. The latter is a model parameter used to produce the realistic appearance of objects. The target dielectric objects to analyze the surface roughness are handcrafted lacquer plates with controlled surface glossiness, as well as several plastics and lacquer products from everyday life. We first define the physical surface roughness as the standard deviation of the surface normal, and provide the computational procedure. We use a laser scanning system to obtain the precise surface height information at tiny flat areas of a surface. Next, a method is developed for estimating the surface roughness parameter based on images taken of the surface with a camera. With a simple setup for observing a glossy flat surface, we estimate the roughness parameter by fitting the Beckmann function to the image intensity distribution in the observed HDR image using the least squares method. A linear relationship is then found between the measurement-based surface roughness and image-based surface roughness. We present applications to glossy objects with curved surfaces.

## 1. Introduction

Most objects with a strong gloss on their surfaces are metals or dielectrics. The light reflection properties of these two types of materials are quite different. Metals are homogeneous objects consisting of specular reflection, while the surface layer of dielectrics is inhomogeneous, and their light reflection is a linear combination of diffuse reflection and specular reflection, known as the dichromatic reflection model [1]. Dielectric objects include many of the everyday items that are familiar to us, such as plastic objects, ceramics, and painted objects [2].

Lacquerware is dielectric, which refers to objects covered with lacquer. Lacquerware includes tableware, containers, furniture, daily necessities, and various small and large objects that people carry around. East Asian countries such as Japan, China, and Korea have a tradition of lacquer crafts that spans thousands of years [3]. The best-known lacquer is a urushiol-based lacquer common in East Asia obtained from the dried sap of Toxicodendron vernicifluum [4].

Lacquerware is basically handmade. Every lacquerware object made with the traditional technique, which has been in use for more than 900 years, is produced using natural wood and urushi lacquer. However, the use of traditional lacquerware has decreased recently because of the increase in cheaper plastic-based lacquerware-like objects. Plastic lacquerware-like objects are so well-made that it is difficult to judge whether an object is plastic or real lacquerware through a casual glance. Unlike natural lacquerware, most synthetic objects are mass-produced through molding by pouring plastic into a mold.

In this paper, we focus on the appearance of glossy inhomogeneous dielectric materials. The overall appearance of three-dimensional (3D) objects results from a combination of the chromatic factor of the surface spectral reflectance and geometric factors such as the surface shape and texture. It is, however, not possible to identify the objects based only on their spectral reflectance and 3D surface shapes, but also the appearance of the objects is affected by the surface microstructure.

The surface roughness is a measure of the microscopic surface structure. It is often quantified by the height deviation along the vertical direction relative to that in the surface shape of an object with an ideal surface. A variety of methods to measure the surface roughness have been proposed for industrial surface inspection [5]. The International Organization for Standardization (ISO) specifies the well-known standard roughness parameter, Ra, in ISO 21920-2:2021 [6]. However, Ra does not necessarily match the perceived appearance of the surface roughness on an object. Instead, the perceived appearance of the surface roughness is correlated with the deviation of the surface normal vectors. Ohtsuki et al. [7] analyzed the surface roughness of human skin, and Oren and Nayar [8] proposed a reflection model for rough surfaces such as concrete and sand in which the surface normals of the surfaces are described using 1D Gaussian distributions.

On the other hand, in the field of computer graphics and image rendering, the concept of roughness has already been used to create a realistic appearance of objects. Almost all surface scattering models and illumination models contain parameters that describe surface roughness. For example, the Beckmann function [9] has often been used to render the realistic appearance of objects [10,11,12,13,14,15]. However, such a roughness is merely a roughness parameter in a mathematical model and does not necessarily represent the actual physical roughness.

In this paper, we study the relationship between the roughness parameter in image rendering and the actual surface roughness—i.e., the physical roughness. We then apply this relationship based on flat surfaces to image rendering and roughness estimation of complex curved surfaces. In order to analyze the surface roughness of glossy objects, we use lacquer plates which are handmade and allow us to control the surface glossiness, as well as several plastics and an actual lacquer product from everyday life. The physical surface roughness of a glossy object surface is defined as the standard deviation of the surface normal.

In the following, in Section 3, we describe a method for estimating surface roughness based on physical measurements. We use a laser scanning system to measure the microscopic surface height of the target object. The surface normal vector is then calculated at every pixel point using the height information.

In Section 4, a method is developed for estimating the roughness parameter based on camera images. We first describe mathematical models for representing specular reflection. The specular lobe generated by light reflection has a significant dependence on the surface roughness, which is approximated using an analytical function with a roughness parameter. We estimate the parameter from the observed high-dynamic-range (HDR) image of a flat surface on the target object.

Section 5 describes a relationship between measurement-based roughness and the image-based roughness parameter. We create a linear regression model between them. The possibility of a linear relationship was shown in our preliminary work using several glossy objects [16]. A reliable model is presented in this paper.

Section 6 presents practical applications to glossy objects. We demonstrate appearance rendering using the estimated roughness parameter, and also estimate the physical roughness values for curved glossy object surfaces.

## 2. Materials Used in the Study

**Figure 1:**
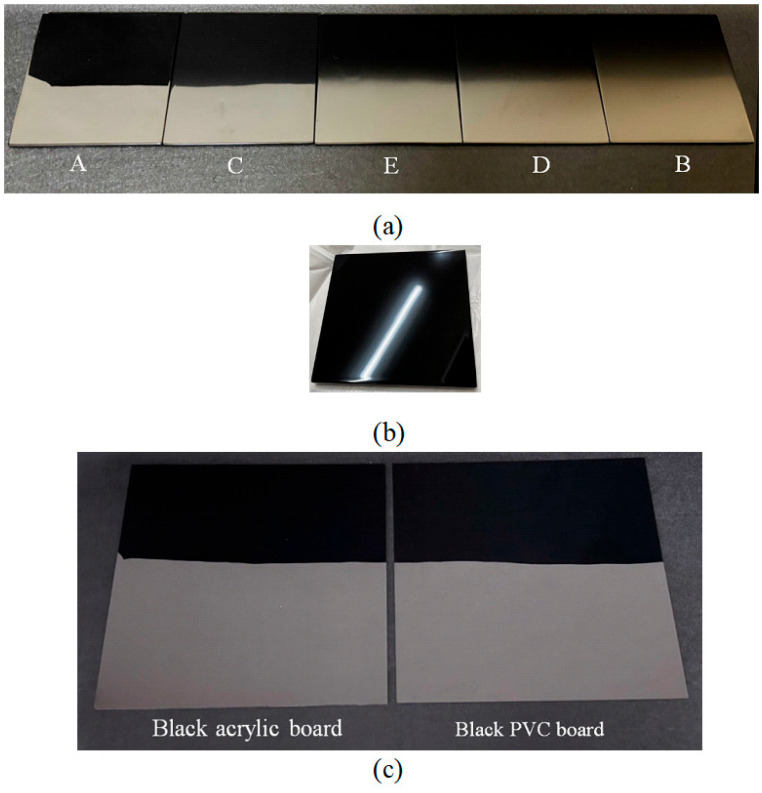

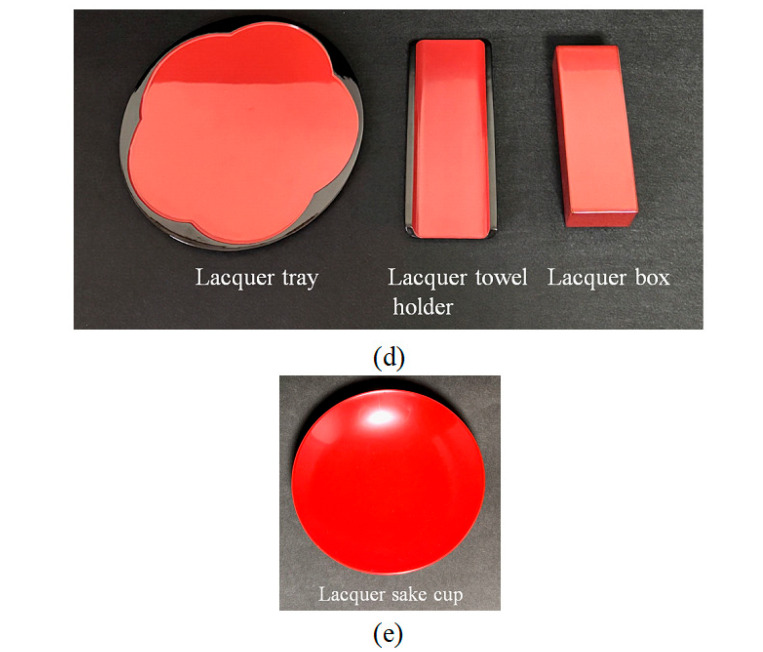
Set of object materials used in this study: (**a**) Black lacquer test plates where the surface gloss levels decrease from left to right. The symbols of A, B,…, E present the order of production based on the finishing process: (**b**) Specular reflection of a ceiling fluorescent light on test sample C: (**c**) Black plastic test board made of acrylic (left) and PVC (right): (**d**) Lacquerware items: tray (left), towel holder (middle), and box (right) selected from everyday items: (**e**) Red Japanese sake cup made of real lacquer.

Figure 1 shows a set of object materials used in this study. Since lacquer products are handcrafted, unlike plastics, it is possible to vary the surface roughness over a wider range, so we used various lacquer products with differing gloss levels. The degree of gloss varies depending on the surface finishing process. Figure 1a shows the five test samples named as follows:

A. Black lacquer, Roiro polished finish,
B. Matte black lacquer painted finish,
C. Glossy painted lacquer finish,
D. 30% glossy black lacquer painted finish, and
E. 50% glossy black lacquer painted finish.

Roiro is a traditional technique where lacquer is applied with a brush, allowed to dry, and then rubbed with charcoal called Suruga charcoal, followed by a process of dozuri, awazuri, and buffing. The surface gloss levels decrease in the order A > C > E > D > B. To illustrate the glossiness of the object surface, Figure 1b presents a specular reflection of a ceiling fluorescent light on test sample C.

Figure 1c shows two black plastic test boards: acrylic on the left and polyvinyl chloride (PVC) on the right. Figure 1d presents three lacquerware samples selected from everyday items: a lacquer tray (left), a lacquer towel holder (middle), and a lacquer box (right). Figure 1e shows a red Japanese sake cup made of real lacquer with a curved surface.

## 3. Measurement-Based Surface Roughness Estimation

### 3.1. Definition and Calculation for Surface Roughness

**Figure 2:**
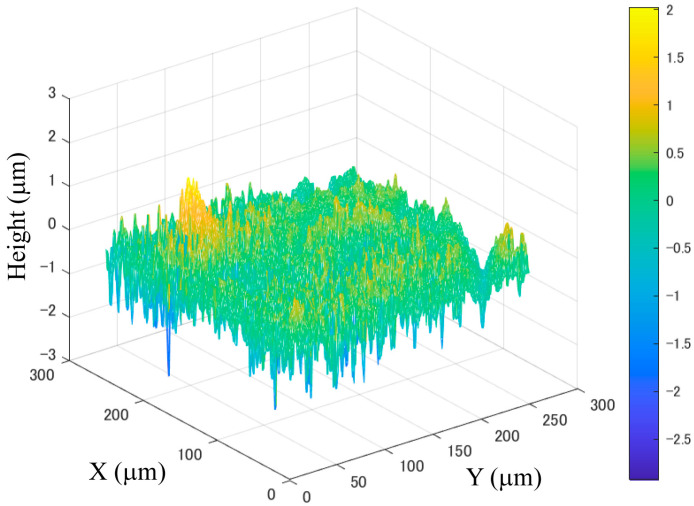
Example of height deviation distribution of an object surface.

The surface roughness of a glossy object’s surface is defined as the standard deviation of the surface normal. The computational procedure is given below [16]. Assume that the height deviation distribution of the surface is obtained at grid points on the XY plane, as shown in Figure 2. The surface normal vector at each grid point can be estimated from the height distribution, as described later.

Let *N* be the total number of surface normal data points. The surface normal vector $n_{i}$ at the *i*-th point (*i* = 1, 2,…, *N*) and the averaged vector $n_{0}$ are described using 3D column vectors:

(1) $$n_{i}=\left[\begin{array}{c} x_{i}\\ y_{i}\\ z_{i} \end{array}\right]\;\;\;\;(i=1,\;2,\;\ldots ,\;N),$$

(2) $$n_{0}=\left[\begin{array}{c} x_{0}\\ y_{0}\\ z_{0} \end{array}\right]\;=\frac{1}{N}\;\sum_{i=1}^{N}n_{i}/\left\|\frac{1}{N}\;\sum_{i=1}^{N}n_{i}\right\|\;,$$

where

(3) $$\left\|n_{i}\right\|=\left\|n_{0}\right\|=1,$$

We define the autocorrelation matrix **T** and the scalar *J* (see [17]) as

(4) $$T=\frac{1}{N}\sum_{i=1}^{N}n_{i}{n_{i}}^{t},$$

(5) $$J=1-{n_{0}}^{t}Tn_{0},$$

where the superscript *t* denotes matrix transposition. We then have

(6) $$\begin{array}{l} J\;=\;\frac{1}{N}\sum_{i=1}^{N}\left(1-{n_{0}}^{t}n_{i}{n_{i}}^{t}n_{0}\right)\;=\;\frac{1}{N}\sum_{i=1}^{N}\left(1-{\left({n_{0}}^{t},n_{i}\right)}^{2}\right)\;\; \end{array},$$

**Figure 3:**
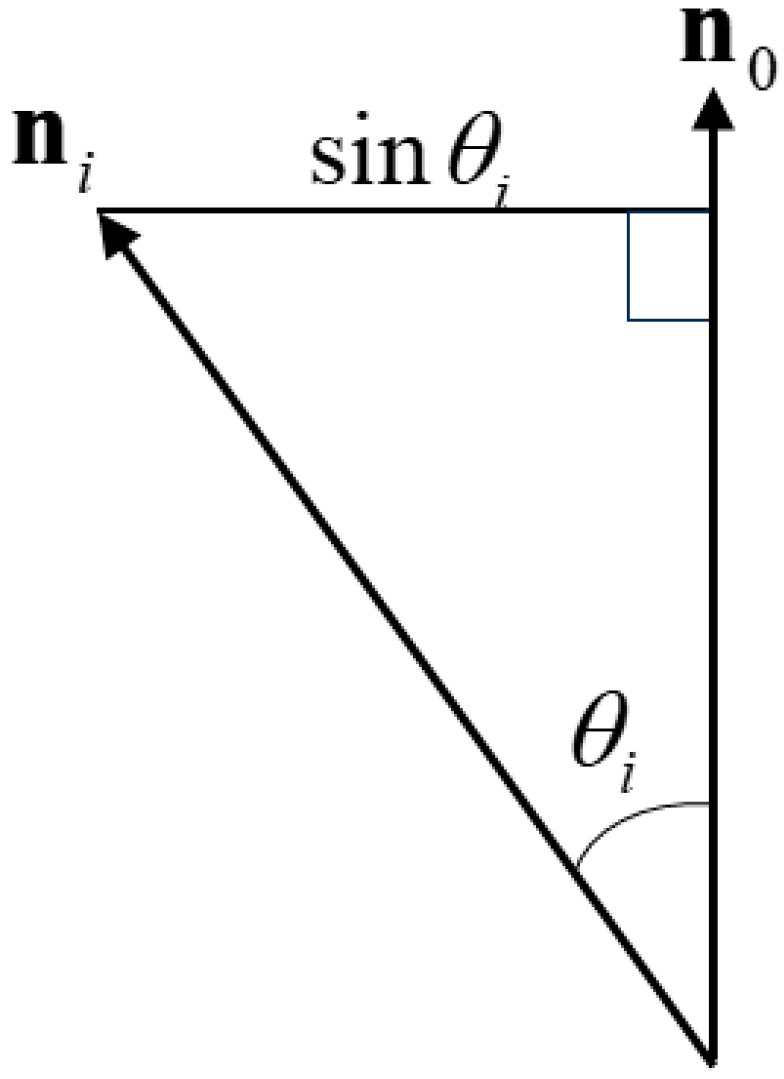
Relationship between the surface normal and average vectors.

Giving the constraints in Equation (3) and the fact that ${n_{0}}^{t}n_{i}$ is the inner product corresponding to the cosine $\cos\theta_{i}$ of the angle $\theta_{i}$ between vectors $n_{0}$ and $n_{i}$, as illustrated in Figure 3. Equation (6) can thus be rewritten as

(7) $$J\;=\;\frac{1}{N}\sum_{i=1}^{N}\left(1-{\left(\cos\theta_{i}\right)}^{2}\right)\;\;=\;\frac{1}{N}\sum_{i=1}^{N}{\left(\sin\theta_{i}\right)}^{2}\;\;,$$

where $\sin\theta_{i}$ is the length of the perpendicular line drawn from the tip of the vector $n_{i}$ to $n_{0}$ (see Figure 3). That is, *J* represents the mean of the squared lengths of the perpendicular lines drawn from each $n_{i}$ to the average vector $n_{0}$. Therefore, the square root of *J*, $\sqrt{J}(≜R)$, is the standard deviation of the surface normal vectors—i.e., the surface roughness *R* defined above.

A procedure of the above calculation is summarized as follows:

Step 1: Averaging the surface normal vectors.

Step 2: Normalizing the vector $n_{0}$.

Step 3: Calculating the autocorrelation matrix **T**.

Step 4: Calculating the scalar *J*.

Step 5: Calculating the standard deviation $R=\sqrt{J}$.

### 3.2. Surface Roughness Estimation Based on Physical Measurement

**Figure 4:**
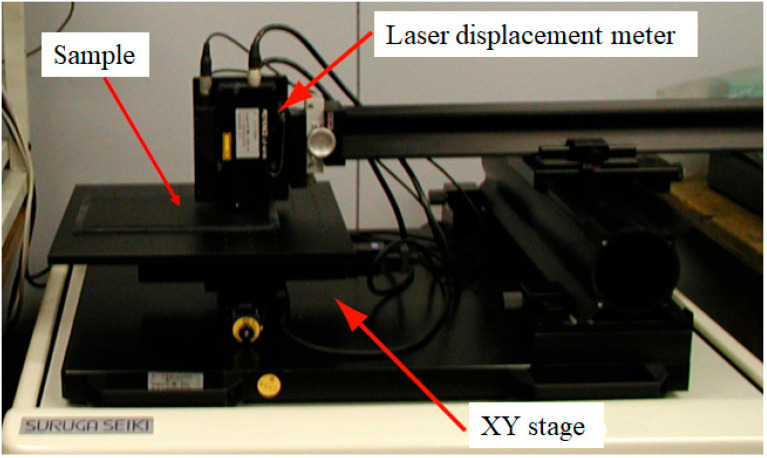
Laser scanning system used in this study.

A laser scanning system, as shown in Figure 4, was used to obtain the precise surface height information of the target objects. The system consisted of an XY-stage and a sensor called a laser displacement meter. The sensor used was mainly the Keyence Model LT8010, and occasionally the Model LT8110. The surface of an object is scanned with high accuracy at a resolution of 0.1 μm. The advantage of this measurement system is that, unlike a camera system, there is no lens distortion owing to the direct measurement of the surface.

Measuring a large area on the surface of the target object is time-consuming and physically challenging, so we measure the height of a small area. As a typical example, the surface height of a tiny flat rectangular area of 0.5 mm × 0.5 mm on the target surface was measured at a 2 μm pitch, resulting in a height profile of 251 × 251 grid points, from which a subset of 201 × 101 points was extracted from the full-size data to avoid noise and analyze height information. Because the target surface was not perfectly flat, the base surface was determined via smoothing using a moving average, and the height deviation was calculated as the difference between the measured and base heights.

**Figure 5:**
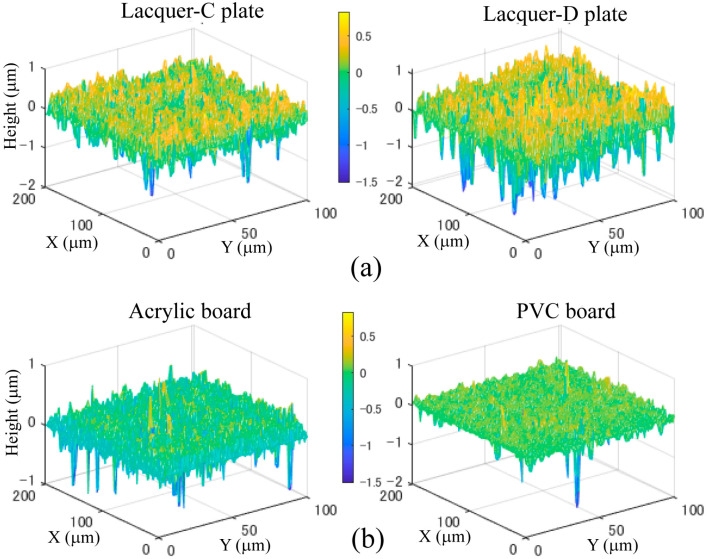
Measured height deviation distributions: (**a**) lacquer-C and -D plates, (**b**) acrylic and PVC plastic boards.

Figure 5 shows the measured height deviation distributions of the four different samples of real lacquer plates and opaque plastic boards, where panels (a) and (b) show the measurement results for the real lacquer-C and -D plates and acrylic and PVC plastic boards, respectively. The *z*-axis scale unit is μm.

**Figure 6:**
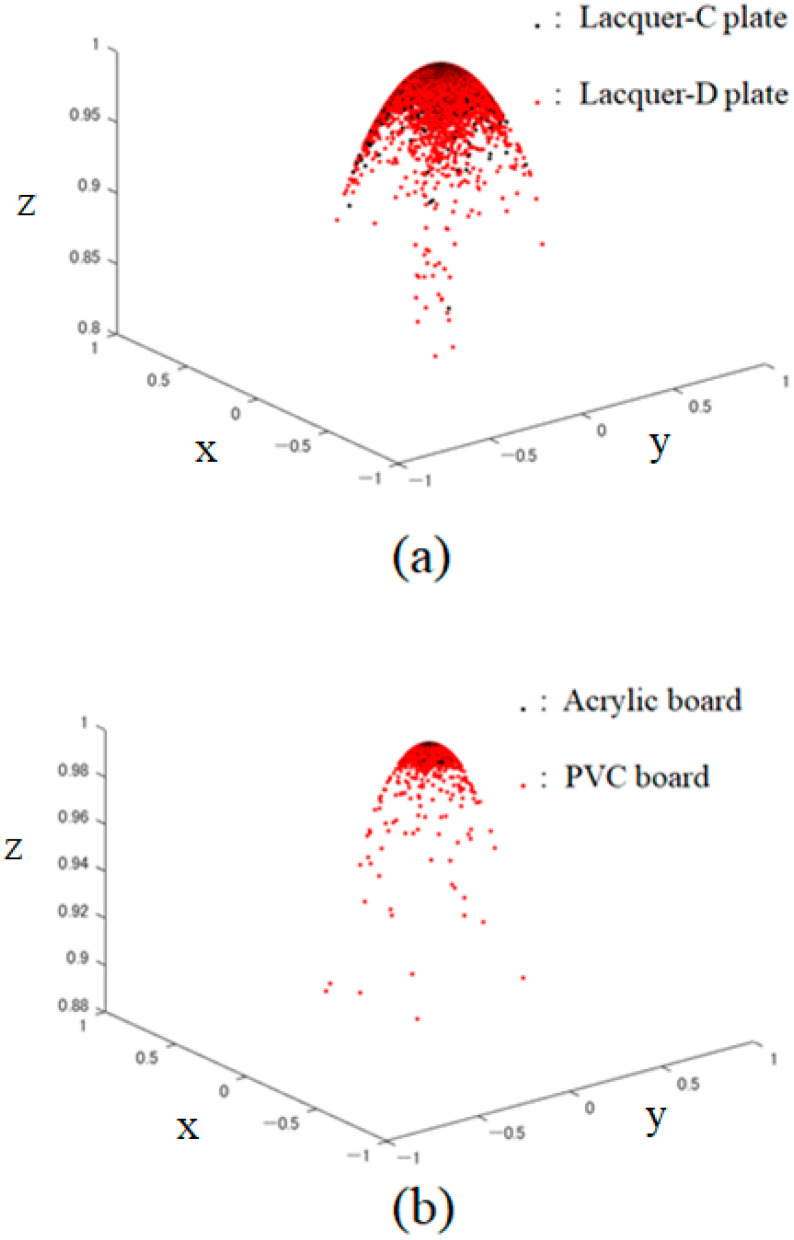
Three-dimensional distributions of the surface normals for the lacquer and plastic objects: (**a**) compare the surface normals between the lacquer-C and -D plates, (**b**) compares the ones between the acrylic and PVC boards.

The MATLAB (R2024b) function “pcnormals” was used to estimate the surface normal vectors from the height data. In this function, the six neighboring points to each point are used for local plane fitting to determine the normal vector at the point [18]. The 3D distributions of the normal vectors suggest that even when object surfaces appear similarly glossy at glance for human vision, the microscopic features of their roughness can be significantly different. In fact, Figure 6 shows the 3D distributions of the surface normals for the lacquer and plastics, where panel (a) compares the surface normals between the lacquer-C and -D plates, and panel (b) compares the ones between the acrylic and PVC boards. Overall, the surface normals of lacquer are more widely distributed than those of plastic, but when examined individually, it can be seen that lacquer C and acrylic have normal directions concentrated more vertically than the others. We should note in Figure 6 that the surface normal vectors are normalized as $x^{2}+y^{2}+z^{2}=1$, so each axis has no physical unit. In the figure, each point is on a unit hemisphere.

**Figure 7:**
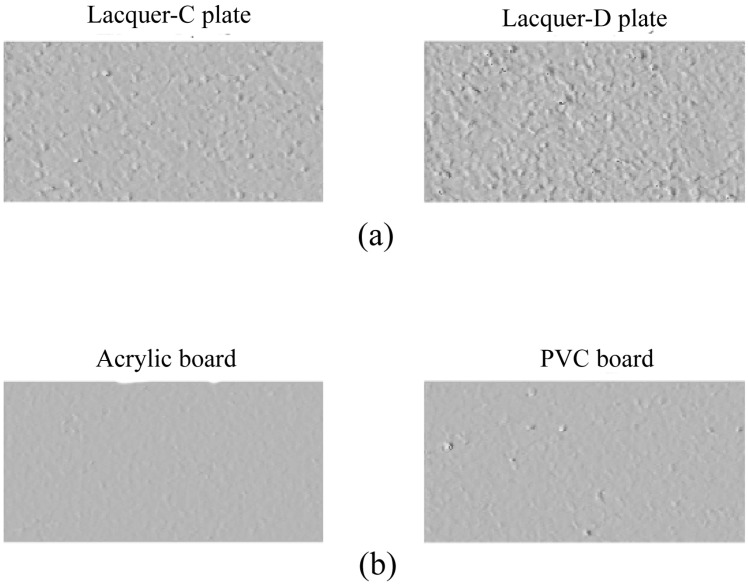
Images shaded using the surface normals over the respective object surfaces: (**a**) lacquer, (**b**) plastic.

Figure 7 shows images shaded using the surface normals obtained at the grid points over the respective object surfaces. The illumination is assumed to be incident at 45°. A comparison of the two sets of images in Figure 7a,b shows that the surface of the real lacquer-D plate looks rougher than that of the plastic objects.

The values of the surface roughness *R* were calculated from the standard deviation of the surface normals according to the previous subsection’s procedure as *R* = 0.0554, 0.1062, 0.0310, and 0.0740 for lacquer-C and -D plates, and acrylic and PVC boards, respectively.

## 4. Image-Based Roughness Parameter Estimation

### 4.1. Mathematical Models for Specular Reflection

The surface roughness parameters of a glossy object are estimated using images captured by a camera. The specular function of a dielectric material is a mathematical function used to model its specular reflection. This function excludes color (spectral) information and primarily represents the geometric characteristics of the specular surface, which depends on the surface orientation, illumination, and viewing direction. Therefore, it can well represent the appearance of a glossy object using parameters related to its roughness and sharpness.

**Figure 8:**
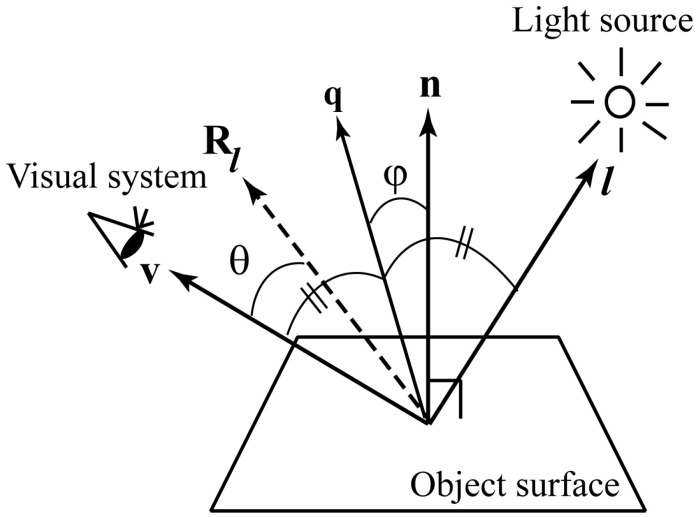
Reflection geometry.

Consider a reflection geometry shown in Figure 8, in which **n** is the surface normal vector, ***l*** the incident light vector, and **v** the viewing vector. Let $R_{l}$ be ***l*** mirrored about **n**, and **q** be the vector bisector of ***l*** and **v**. Specular reflection occurs only within a limited range of viewing angles. This reflection component is typically strongest along the direction of reflection $R_{l}$ and decreases rapidly as the angle θ between $R_{l}$ and **v** increases. This rapid falloff is often approximated as

(8) $$f_{\mathrm{PC}}(\theta )=\;\beta \;\cos^{m}\theta ,$$

where β is a constant representing the specular peak intensity, and *m* is a parameter representing the surface roughness. This intensity distribution is known as the Phong distribution [10]. If the highlight has a pointed peak, a Gaussian distribution can be used to model the sharp falloff as follows:

(9) $$f_{\mathrm{PG}}(\theta )=\;\beta \exp\left(-m\theta^{2}\right),$$

However, simple specular functions such as the Phong distribution cannot describe the surface specularity of rough surfaces adequately due to the presence of unknown constant parameters. In addition, there is always some surface roughness even when the surface appears smooth. The shape of the specular reflection lobe generated by dichromatic reflection depends significantly on the surface roughness. Rough specular surfaces are often modeled as assemblies of small planar patches, known as microfacets [19].

The Beckmann distribution is a physics-based microfacet distribution model [9]. The specular lobe can be approximated using the Beckmann distribution function with a surface roughness parameter *m* as

(10) $$f_{B}(\varphi )=\;\left(\frac{1}{m^{2}\cos^{4}\varphi }\right)\;\exp\left\{-{\left(\tan\varphi /m\right)}^{2}\right\},$$

where φ is the angle between **q** and **n**. Unlike the empirical model in Equations (8) and (9), this function yields the absolute magnitude of the reflectance without the need for arbitrary constants. The Beckmann function is a more accurate function than the Gaussian for micro-surface [19]. The distribution function called GGX is also a distribution function that models microfacets, similar to the Beckmann function [20]. This function is written as

(11) $$f_{G}(\varphi )=\frac{m^{2}}{\cos^{4}\varphi {\left(m^{2}+\tan^{2}\varphi \right)}^{2}}\;.$$

It is shown that, when the gloss is strong, there is not much difference between the Beckmann and GGX distributions [20]. The GGX distribution is equivalent to a microfacet distribution that Trowbridge and Reitz introduced in 1975 [21]. Our experimental results suggested that the Beckmann function performed slightly better than GGX, so we adopted the Beckmann function in this paper.

### 4.2. Roughness Parameter Estimation Based on Camera Images

**Figure 9:**
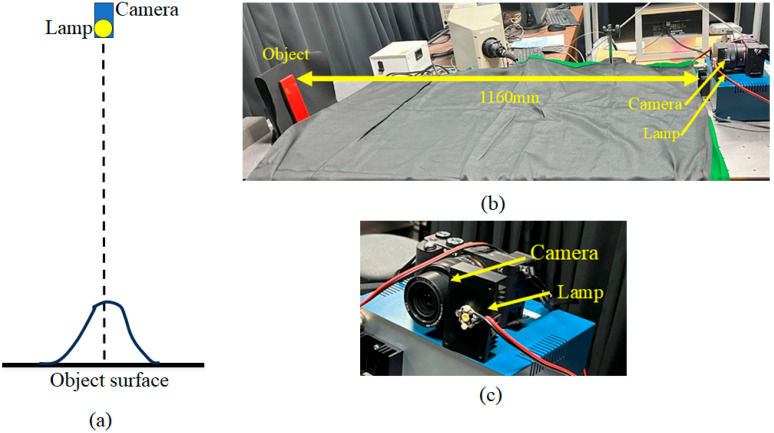
Setup for capturing a glossy flat object surface: (**a**) overall setup, (**b**) actual placement of the object, camera, and LED lamp, (**c**) close-up view of the camera and LED lamp.

To estimate the Beckmann roughness parameter from camera images, we consider a simple measurement setup. Figure 9 illustrates the setup for capturing a glossy flat object surface. The light source and camera are positioned above the surface, and the surface reflection forms a 1D intensity distribution curve. In this case, the angles φ and θ in Figure 8 are both equal to the viewing angle. In the actual system we used, the distance between the object and the light source (camera) was 1160 mm, and the light source was a small LED. A digital single-lens reflex (DSLR) camera (Sony alpha 7C) with a 14-bit depth per color channel was used. The camera images were captured in the lossless SONY-ARW raw image format. The captured images of the object surface include strong glosses or highlights. Many images were captured by changing the shutter speed and then linearly combined to obtain an image without saturation and with the highest dynamic range to create an HDR image of the target surface.

**Figure 10:**
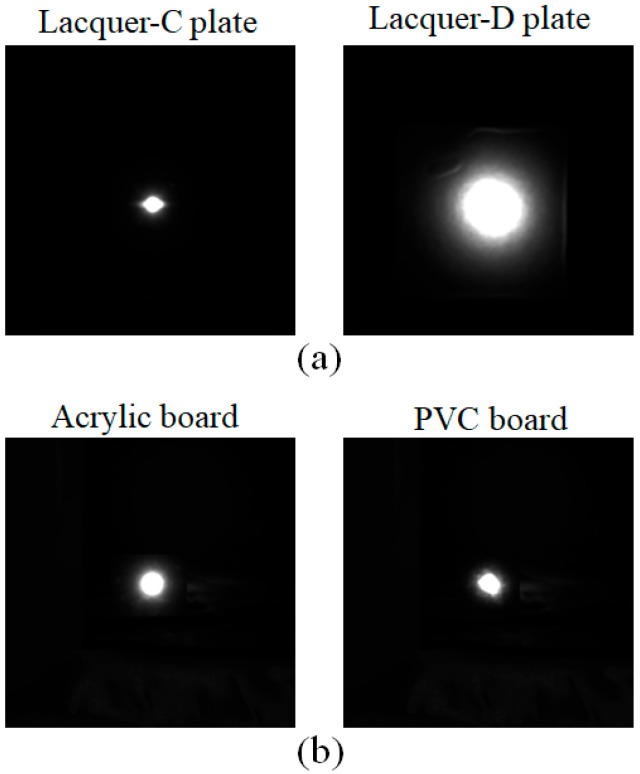
HDR images obtained from the lacquer-C and -D plates in panel (**a**) and acrylic and PVC plastic boards in panel (**b**), respectively.

**Figure 11:**
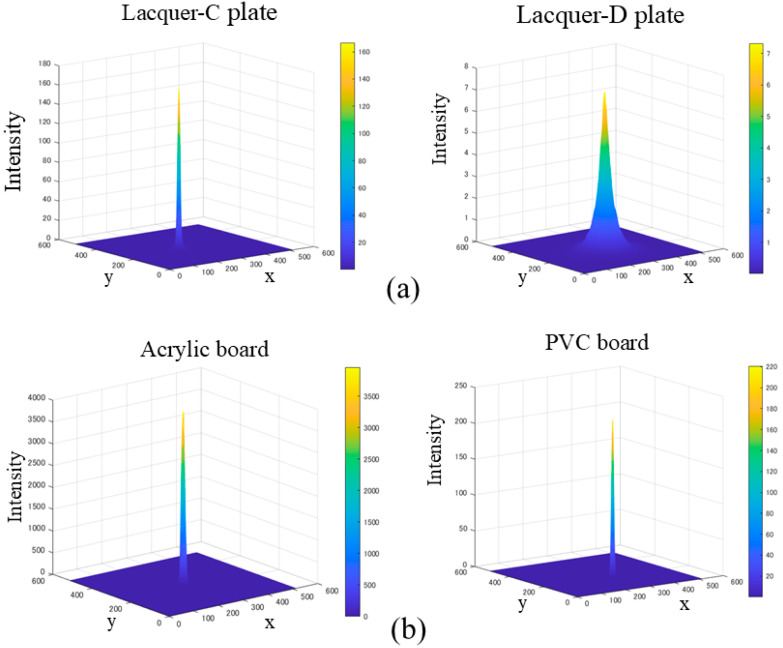
Mesh representations of the luminance intensity distributions for (**a**,**b**).

Figure 10 shows the HDR images obtained from the same objects as the previous section, where panels (a) and (b) are the HDR images for the lacquer-C and -D plates and acrylic and PVC plastic boards, respectively. The image intensity is a relative value, normalized using a white reference standard with a matte surface photographed under the same conditions. To make the intensity distribution of the HDR image easier to understand, we draw a 2D histogram of the image. Figure 11 shows the corresponding mesh representations of the luminance intensity distributions for (a) and (b). The mesh size is 512 × 512, and one pixel corresponds to approximately 0.0246°. The 2D intensity distribution is isotropic in the (x, y) plane and symmetric about each axis. Therefore, the Beckmann function in Equation (10) can be fitted to the 1D profile of the intensity distribution.

We employed the least-squares fitting method. Let I(φ) and $f_{B}(\varphi )$ be the 1D image intensity and the Beckman function, respectively, at the viewing angle φ. The roughness parameter *m* is treated as a variable included in the function in the form of m=1/i    (i=1, 2, …). We search for *m* that minimizes the squared error

(12) $$E={\sum_{\varphi }\left(I(\varphi )-f_{B}(\varphi )\right)}^{2}$$

We should note that in the method of m=1/i    (i=1, 2, …), when *m* is small (i.e., when *i* is large), such as on glossy surfaces, the step size becomes smaller, so it is more accurate than when the step size is uniform, such as m=0.001×i   (i=1, 2, …).

**Figure 12:**
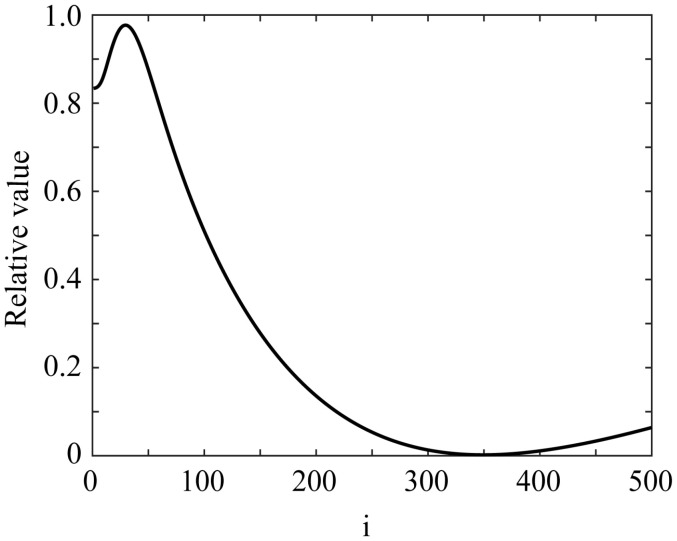
Error variation as a function of *i* for C plate.

**Figure 13:**
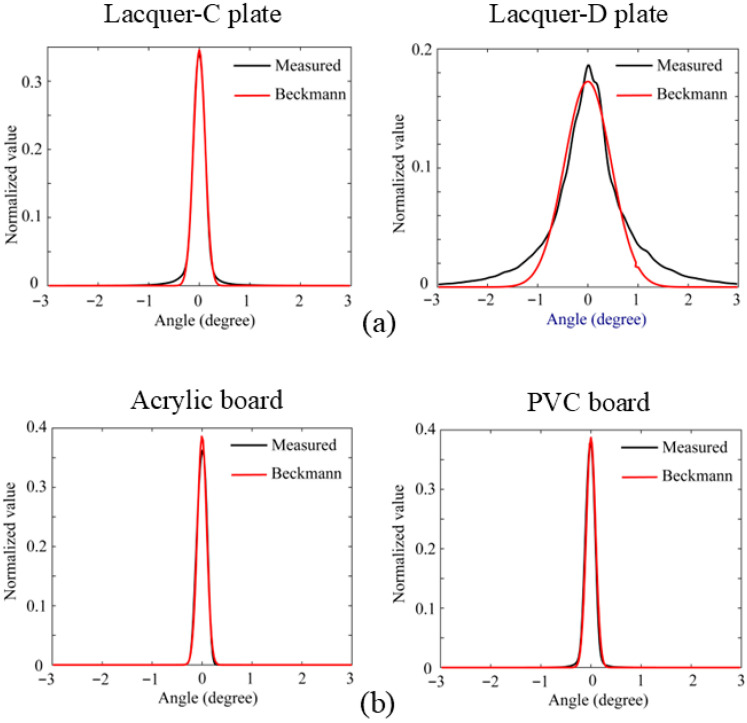
Fitting results for the four glossy objects as functions of the viewing angle φ, where the black and red curves represent the intensity curve of the HDR image and the fitted Beckmann function, respectively: (**a**) lacquer, (**b**) plastic.

Figure 12 draws the error variation as a function of *i* for the C plate, with the minimum error E = 0.001997 occurring at *i* = 349, corresponding to *m* = 0.002865. Figure 13 shows the fitting results for the four glossy objects as functions of the viewing angle φ, where the black and red curves represent the intensity curve of the HDR image and the fitted Beckmann function, respectively. Thus, the roughness parameters *m* were estimated as *m* = 0.00287, 0.011236, 0.002288, and 0.002222 for lacquer-C and -D plates, and acrylic and PVC boards, respectively. It can be seen that the HDR intensity curves observed by the camera closely match the Beckmann distribution with a single parameter, particularly for the lacquer-C plate, and the acrylic and PVC boards.

## 5. Relationship Between Measurement- and Image-Based Roughness

**Figure 14:**
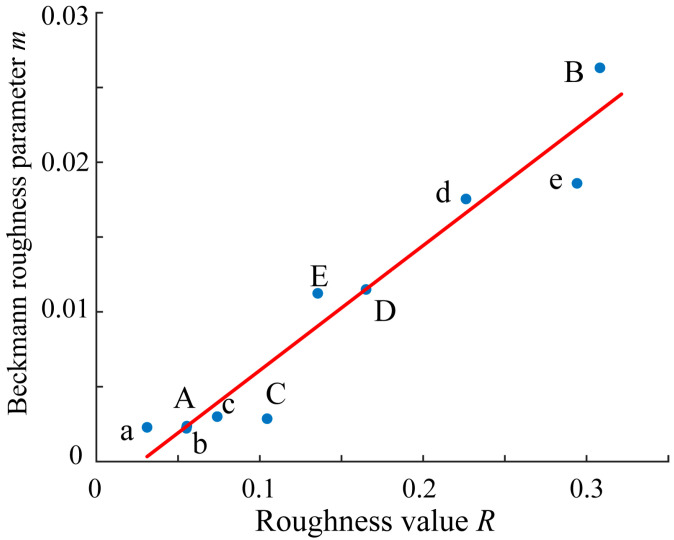
Coordinate plots of the estimated *R* and *m* values in a two-dimensional coordinate system (*R*, *m*) for ten objects, where the symbols demote the following: A–E: black lacquer plates in Figure 1a, a: black acrylic board, b: black PVC board, c: lacquer tray, d: lacquer box, and e: lacquer tower holder.

For all the glossy objects with flat surfaces in Figure 1, we estimated the surface roughness *R* by the physical measurement using a laser scanning system, and also estimated the parameter *m* to mathematically model the surface roughness based on HDR images taken by a camera. Figure 14 plots the estimated *R* and *m* values for ten objects in a two-dimensional coordinate system (*R*, *m*), where the symbols denote the following: A–E: black lacquer plates in Figure 1a, a: black acrylic board, b: black PVC board, c: lacquer tray, d: lacquer box, and e: lacquer tower holder.

The correlation coefficient *r* between each *R* and *m* element pair was calculated as *r* = *corr*(*R*, *m*) = 0.9655, and also the coefficient of determination of the regression Equation (13) was $r^{2}=0.9322$ (see [22]). These results suggest a strong correlation between the measurement-based roughness values and the image-based Beckmann roughness parameters, and the latter is well explained by the former to Equation (13).

(13) $$m=c_{0}\;+\;c_{1}R.$$

The coefficients $c_{0}$ and $c_{1}$ were determined by least-squares fitting to the ten estimated pairs as $c_{0}$ = −0.0022 and $c_{1}$ = 0.1166 Note that $c_{0}$ represents a small bias term.

## 6. Practical Applications to Glossy Objects

### 6.1. Appearance Reproduction Using the Estimated Roughness Parameter

The Cook–Torrance model [11] was employed to reproduce the realistic appearance of glossy objects under various conditions. The spectral reflection model for the color signal, incorporating the light source and reflectance, is given by

(14) $$C(n,v,l,\lambda )\;=\;\left(n•l\right)S_{d}(\lambda )E(\lambda )+\;cF_{0}\frac{f(\varphi )G\left(n,v,l\right)}{\left(n•v\right)\left(n•l\right)}S_{p}(\lambda )E(\lambda ),$$

where the first and second terms in the right-hand side represent the diffuse and specular reflection components, respectively, and *c* is a constant, E(λ) the spectral power distribution of the light source, $S_{d}(\lambda )$ the spectral reflectance for the diffuse reflection component, $S_{p}(\lambda )$ the spectral reflectance for the specular reflection component, $F_{0}$ the Fresnel reflectance at normal incidence, f(φ) the Beckmann distribution function in Equation (10), and *G,* the geometric attenuation factor describing self-shadowing due to the microfacets (see [11]), the symbol • the inner product of two vectors.

We used the physical spectral renderer Mitsuba (Version 0.5.0) [23] to predict the reflection based on an underlying Monte Carlo simulation. The Cook–Torrance model was implemented in the renderer by Guarnera [15]. The reflectance and illuminance spectral functions were represented in 5 nm intervals within the wavelength range of 400–700 nm. A perspective camera model was used to set the field of view such that the rendered image fitted the acquired image. The location and orientation of the camera and lighting were adjusted to match the actual camera images. An output image with a size of 512 by 512 pixels was spectrally rendered. The 3D shape data of the objects acquired using a 3D scanner were input as OBJ files. The spectral reflectance $S_{d}(\lambda )$ and illuminant distribution E(λ) from the target object and the actual light source were measured using a spectral colorimeter and a spectral radiometer, respectively. The specular reflectance $S_{p}(\lambda )$ was set to 1. The attenuation factor *G* could be regarded as 1 (no shadowing) in the usual observation condition for a smoothed surface. The color signal *C* obtained at each pixel was converted to the tristimulus values XYZ and then to sRGB.

**Figure 15:**
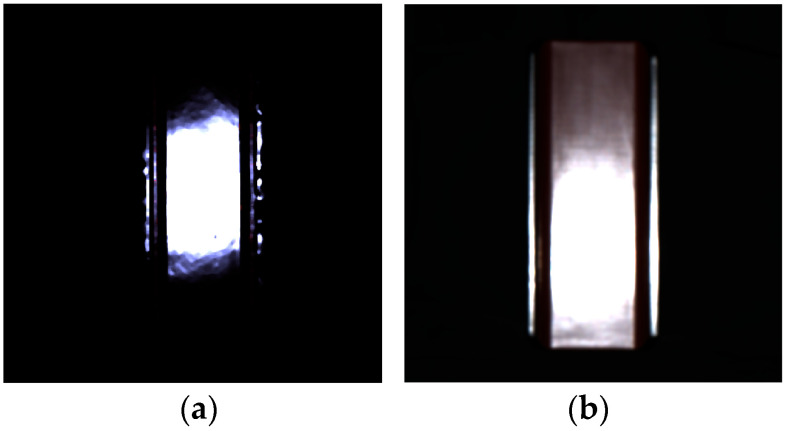
Rendered image (**a**) and real image (**b**) of the lacquer towel holder.

**Figure 16:**
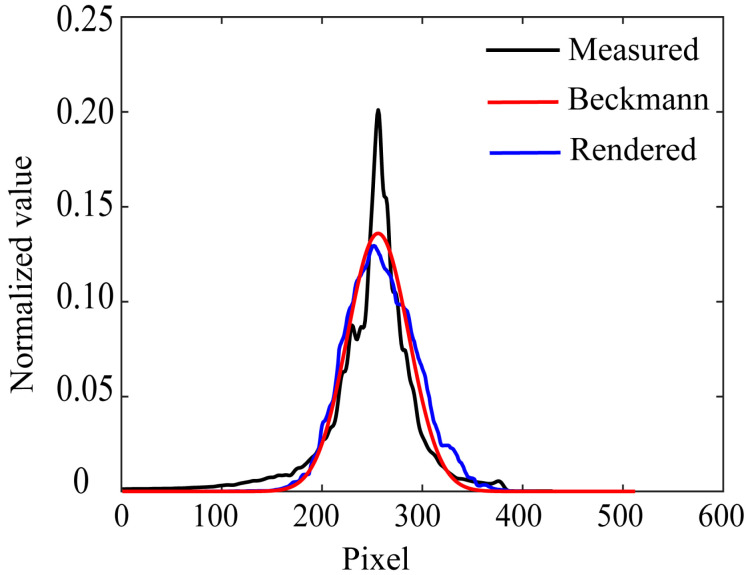
Comparison among three curves of the measured intensity distribution obtained from the original HDR image, the fitted Beckmann distribution, and the rendered image intensity distributions.

We first verify the reliability of the estimated roughness parameters in the rendered images. Figure 15a shows the rendered image of the lacquer towel holder using Mitsuba based on the setup shown in Figure 9. The estimated roughness parameter *m* = 0.01859 was used. The light source was assumed to be a point light source. Figure 15b shows the real image for comparison. We calculated the intensity profile around the specular peak in the rendered image and compared it with the measured intensity and the fitted Beckmann function. Figure 16 compares three curves of the measured intensity distribution obtained from the original HDR image, the fitted Beckmann distribution, and the rendered image intensity distribution. The Beckmann distribution was estimated by the least squares fitting to the measured intensity distribution, and it accurately represents the rendered image’s intensity distribution.

Let us consider a curved object surface. If the surface is gently curved, it is possible to measure the surface heights in small, approximately planar regions to estimate the surface roughness. However, because the specular profile of a glossy object reflects the light reflection at the entire object, the intensity distribution of a curved surface cannot be directly obtained from the captured images, unlike in flat surfaces, to estimate the surface roughness parameter in the Beckmann function.

**Figure 17:**
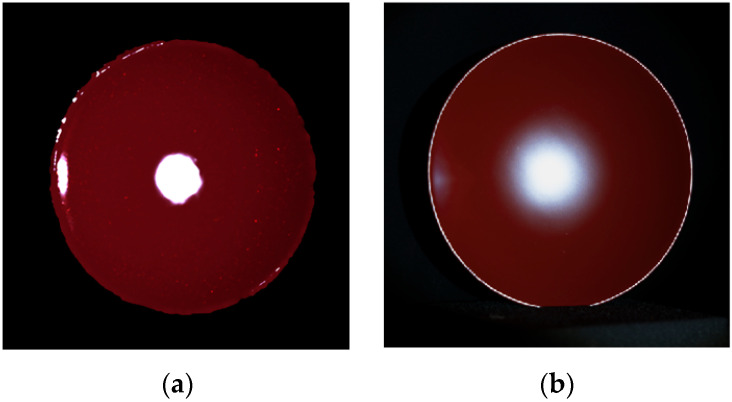
Rendered image (**a**) using the estimated roughness parameter from the physically measured roughness value and real image (**b**) of the lacquer sake cup.

Look at the lacquer sake cup in Figure 1e. The bottom of the cup is flat locally. Therefore, according to the measurement and computation procedure in Section 3, the surface roughness R was estimated as *R* = 0.1848. The roughness parameter *m* in the Beckmann function was subsequently estimated as *m* = 0.0193 using the regression model in Equation (13). Figure 17a shows the rendered image of the lacquer sake cup using the estimated roughness parameter in the Cook–Torrance mode, assuming the camera is placed 500 mm from the object and tilted 15 degrees from the normal, and the LED light, regarded as a point light source, is placed 1300 mm away and tilted 15 degrees in the opposite direction. Figure 17b shows the real image for comparison. The rendered image does not look similar to the actual image of the cup. This behavior is due to the falloff at the tails of the Beckmann distribution, which is too sharp and cannot perfectly fit highly polished materials such as high-gloss surface.

### 6.2. Estimation of Physical Roughness for Glossy Object with Curved Surface

The measurement-based surface roughness estimation method using a laser scanning system is primarily applicable to relatively flat and thin surfaces. On the other hand, if 3D shape data for a curved object is available, it is possible to vary the roughness parameter *m* of the Beckmann function, create many glossy surfaces by rendering, and predict the parameter that gives a surface appearance close to that of the glossy surface captured by a camera. Specifically, we can find a parameter that makes the intensity distribution around the specular peak closer to the actual image intensity. The physical surface roughness *R* can then be estimated from the regression equation.

**Figure 18:**
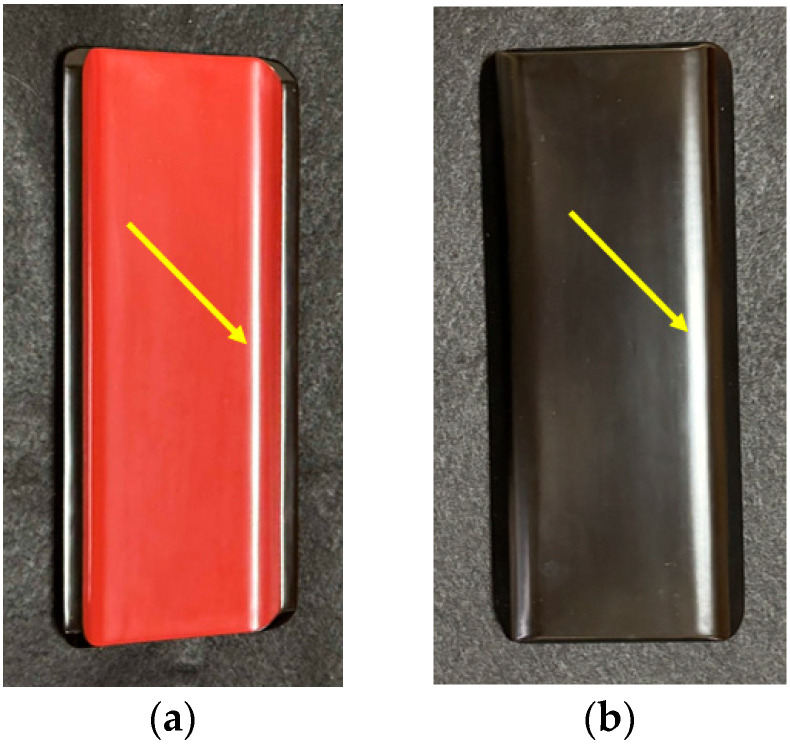
Examples of glossy curved surfaces, where (**a**) is an image taken with the front surface of the lacquer towel holder by tilting the object, and (**b**) is an image taken with the back surface in the same condition.

Examples of glossy curved surfaces are demonstrated in Figure 18. Figure 18a is an image taken with the front surface of the lacquer towel holder by tilting the object, where gloss appears in the grooves on the concave surface as indicated by the yellow arrow. Figure 18b is an image taken with the back surface in the same condition, where gloss appears on the convex surface. To precisely render the specular reflections, we used the rough dielectric model in Mitsuba, where the Beckmann function was used to model the surface roughness.

**Figure 19:**
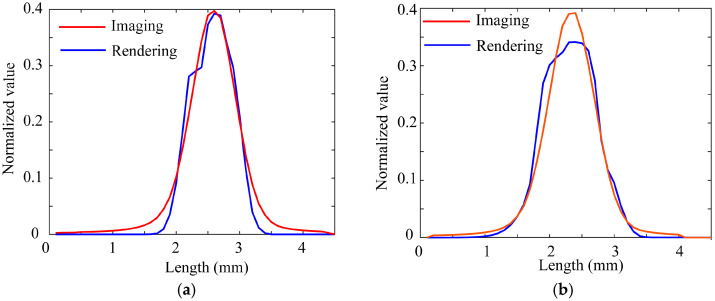
Comparison between the intensity distributions obtained from the captured HDR images and those of the fitted rendered images, where (**a**,**b**) correspond to the front and back surfaces.

Figure 19a,b compare the intensity distributions around the specular peaks obtained from the directly captured HDR images and those of the fitted rendered images, where (a) and (b) correspond to the front and back surfaces. The intensity distributions are taken orthogonally to the highlight lines. The camera and light were supposed to be 300 mm away from the object and 1160 mm away, respectively. The fitting results suggested that *m* = 0.030 for (a) and *m* = 0.025 for (b). The physical surface roughness was finally estimated as R = 0.2762 for (a) and R = 0.2333 for (b) from Equation (13).

## 7. Conclusions

In this paper, we have studied the relationship between the surface roughness of glossy object surfaces on dielectric objects, such as painted objects and plastics—specifically, the physical surface roughness and the roughness parameter used in image rendering. It should be noted that even a mirror-like surface on a dielectric object exhibits microscopic irregularities. The physical surface roughness refers to a measure of the microscopic surface structure of a real object’s surface. The image-based surface roughness parameter is a modeling factor used to produce the realistic appearance of objects in computer graphics and image rendering. To analyze the surface roughness of glossy dielectric objects, we used handcrafted lacquer plates with controlled gloss levels, as well as several plastics and an actual lacquer product from everyday life.

First, we defined the physical surface roughness of a glossy object surface as the standard deviation of the surface normal, and described the computational procedure from the surface height information. A laser scanning system was used to obtain the precise surface height information of the target objects. By scanning tiny flat rectangular areas on the surface, we were able to measure the surface heights precisely on a dense grid of points.

Next, a method was developed for estimating the surface roughness parameter based on images taken of the surface with a camera. We adopted the Beckmann distribution function with a roughness parameter, which was used to model the microfacet distribution describing the specular surface with a set of microfacets. We created a simple setup for capturing a glossy flat object surface. The roughness parameter was estimated by fitting the Beckmann distribution function to the image intensity distribution in the observed HDR image of a target flat surface in the least squares method.

Based on the above results, we demonstrated the relationship between measurement-based surface roughness and image-based surface roughness, and proposed a linear regression model to describe the relationship. Furthermore, we presented practical applications to glossy objects with curved surfaces using the relationship. The measurement-based method using a laser scanning system is primarily suitable for relatively thin and flat object surfaces. If the surface is gently curved, the surface heights can be measured in small, approximately planar areas to estimate the surface roughness. However, 3D shape data are required for general curved surfaces. Under this condition, we proposed a method to identify the roughness parameter that produces a rendered intensity distribution most closely matching the actual image intensity distribution, and then estimate the physical surface roughness from a regression model equation.

In this paper, we used Mitsuba (Version 0.5.0) and calculated roughness parameters directly from captured images. A differentiable renderer makes it possible to automatically optimize scene parameters to match a reference photograph using the numerical derivatives. Although the latest version of Mitsuba (Version 3) includes differentiable rendering, the support in Mitsuba 3 for spectral rendering is very limited. Roughness parameter estimation using the differentiable rendering is left as future work.

As mentioned in Section 6.1, the target object was highly glossy, so the rendered image did not perfectly match the actual photographed image of the object. The improvement of rendering accuracy remains future work. We studied mainly the roughness estimation of flat object surfaces. The roughness estimation of arbitrary shape surfaces, including curved surfaces, and solving numerical optimization to find the minimum point for the roughness parameter are future challenges.

## Data Availability

The datasets presented in this article are not readily available because the data are part of an ongoing study.

## References

[1] Tominaga S. (1994). Dichromatic Reflection Models for a Variety of Materials. Color Res. Appl..

[2] Tominaga S. (1991). Surface identification using the dichromatic reflection model. IEEE Trans Pattern Anal. Mach. Intel..

[3] https://en.wikipedia.org/wiki/Lacquerware.

[4] https://en.wikipedia.org/wiki/Toxicodendron_vernicifluum.

[5] Shao M.Q., Xu D., Li S.-Y., Zuo X.-G., Chen C.-K., Peng G.-Z., Zhang J.-M., Wang X.-C., Yang Q. (2023). A review of surface roughness measurements based on laser speckle method. J. Iron Steel Res. Int..

[6] (2021). Specifications, Geometrical Product. “Surface Texture: Profile—Part 2: Terms, Definitions and Surface Texture Parameters”.

[7] Ohtsuki R., Sakamaki T., Tominaga S. (2013). Analysis of skin surface roughness by visual assessment and surface measurement. Opt. Rev..

[8] Oren M., Nayar S. (1995). Generalization of the Lambertian model and implications for machine vision. Int. J. Comput. Vis..

[9] Beckmann P., Spizzichino A. (1963). The Scattering of Electromagnetic Waves from Rough Surfaces.

[10] Phong B.T. (1975). Illumination for computer-generated pictures. Comm. ACM.

[11] Cook R.L., Torrance K.E. (1982). A reflection model for computer graphics. ACM Trans. Graph..

[12] Ribardière M., Meneveaux D., Bringier B. Simonot Appearance of interfaced Lambertian microfacets using STD distribution. Proceedings of the Workshop on Material Appearance Modeling.

[13] Lee J.H., Jarabo A., Jeon D.S., Gutierrez D., Kim M.H. (2018). Practical multiple scattering for rough surfaces. ACM Trans. Graph..

[14] Chermain X., Claux F., Mérillou S. (2020). A microfacet-based BRDF for the accurate and efficient rendering of high-definition specular normal maps. Vis. Comput..

[15] Tominaga S., Guarnera G.C. Measuring, modeling, and reproducing material appearance from specular profile. Proceedings of the Color and Imaging Conference (CIC27).

[16] Tominaga S., Doi M. Surface roughness estimation for reproducing appearance of glossy object surfaces. Proceedings of the Color and Imaging Conference (CIC32).

[17] Fisher N.I., Lewis T., Embeton B.J.J. (1987). Statistical Analysis of Spherical Data.

[18] MATLAB Pcnormals. https://jp.mathworks.com/help/vision/ref/pcnormals.html?lang=en.

[19] Hall R. (1989). Illumination and Color in Computer Generated Imagery.

[20] Walter B., Marshner S.R., Li H., Torrance K. Microfacet models for refraction through rough surface. Proceedings of the 18th Eurographics Symposium on Rendering.

[21] Atanasov A., Koylazov V., Dimov R., Wilkie A. Microsurface transformations. Proceedings of the Eurographics Symposium on Rendering 2022.

[22] Ross S.M. (2004). Introduction to Probability and Statistics for Engineers and Scientists.

[23] https://www.mitsuba-renderer.org.

